# Tolerance and Cross-Tolerance following Toll-Like Receptor (TLR)-4 and -9 Activation Are Mediated by IRAK-M and Modulated by IL-7 in Murine Splenocytes

**DOI:** 10.1371/journal.pone.0132921

**Published:** 2015-07-28

**Authors:** Mark W. Julian, Heather R. Strange, Megan N. Ballinger, Richard S. Hotchkiss, Tracey L. Papenfuss, Elliott D. Crouser

**Affiliations:** 1 Dorothy M. Davis Heart and Lung Research Institute, Division of Pulmonary Allergy, Critical Care, and Sleep Medicine, Wexner Medical Center, Columbus, OH, United States of America; 2 College of Veterinary Medicine, Department of Veterinary Biosciences, The Ohio State University, Columbus, OH, United States of America; 3 Departments of Anesthesiology, Medicine and Surgery, Washington University School of Medicine, St. Louis, MO, United States of America; University of Medicine and Dentistry of New Jersey—New Jersey Medical School, UNITED STATES

## Abstract

**Objective:**

Immune suppression during critical illness predisposes to serious infections. We sought to determine the mechanisms regulating tolerance and cross-tolerance to common pro-inflammatory danger signals in a model that recapitulates the intact *in vivo* immune response.

**Materials and Methods:**

Flt3-expanded splenocytes obtained from wild-type or matching IRAK-M knockout (IRAK-M-/-), C57BL/6, male mice (8–10 weeks old) were treated repeatedly or alternately with either LPS or CpGA DNA, agonists of Toll-like receptor (TLR)-4 and -9, respectively, over successive 24-hour periods. Supernatants were collected following each 24-hour period with cytokine release (ELISA) and splenocyte IRAK-M expression (Western blot) determined. Tolerance and cross-tolerance were assessed in the absence or presence of programmed death receptor (PD)-1 blocking antibody or IL-7 pre-treatment.

**Main Results:**

Splenocytes notably exhibited both tolerance and cross-tolerance to subsequent treatments with either LPS or CpGA DNA. The character of tolerance and cross-tolerance in this model was distinct following initial LPS or CpGA treatment in that TNFα and IFNγ release (not IL-10) were suppressed following LPS; whereas, initial CpGA treatment suppressed TNFα, IFNγ and IL-10 release in response to subsequent stimulation (LPS or CpGA). Tolerance and cross-tolerance were unrelated to IL-10 release or PD-1 but were attenuated in IRAK-M-/- splenocytes. IL-7 significantly suppressed IRAK-M expression and restored TNFα and IFNγ production without influencing IL-10 release.

**Conclusions:**

In summary, acute immune tolerance and cross-tolerance in response to LPS or CpGA were distinct in that LPS selectively suppressed pro-inflammatory cytokine responses; whereas, CpGA suppressed both pro- and anti-inflammatory responses. The induction of tolerance and cross-tolerance in response to common danger signals was mechanistically unrelated to IL-10 or PD-1 but was directly influenced by IRAK-M expression. IL-7 reduced IRAK-M expression and attenuated immune tolerance induced by either LPS or CpGA, and thus may be useful for reversal of immune tolerance in the setting of critical illness.

## Introduction

Tolerance in the setting of immune responses refers to a state of refractoriness towards a second stimulation by an immunostimulatory agent. The most extensively studied example of immune tolerance is in response to lipopolysaccharide (LPS), a component of Gram-negative bacteria which promotes immune cell signal transduction through Toll-like receptor (TLR)-4, wherein low-level pre-treatment with LPS is shown to induce hyporesponsiveness to subsequent LPS exposure *in vitro* [1] and *in vivo* [2–4]. LPS also promotes cross-tolerance to CpG- containing DNA [5], which is a putative immunostimulatory component of common DNA-viruses [(e.g., adenovirus, parvovirus, herpes simplex virus (HSV), and cytomegalovirus (CMV)] and mitochondrial DNA that is recognized by TLR-9 [6–11].

The mechanisms underlying tolerance induction and sustained cellular hyporesponsiveness remain unclear. Reductionist models (e.g., cell lines, immune cell isolates) fail to replicate *in vivo* immune tolerance and cross-tolerance. For example, CpG DNA treatment of RAW264.7 macrophages induces cross-tolerance to subsequent challenge by LPS [12]. In contrast, low-dose CpG DNA pre-treatment *in vivo* selectively protects against subsequent CpG DNA challenge and paradoxically enhances TNFα production and organ damage in response to subsequent LPS challenge [5]. Whereas alterations of several critical signal transduction pathways, particularly those regulated by IRAK-1 and IRAK-M, are implicated in the induction of immune cell tolerance [13, 14], the situation is likely to be much more complex *in vivo*. The intact immune response depends upon interactions among multiple cell types, and immune tolerance *in vivo* is potentially influenced by direct intercellular interactions, including inhibitory effects of programmed death receptor-1 (PD-1) and programmed death-ligand 1 (PD-L1) [15], and indirect suppression of nearby and remote immune cells by immune-modulating cytokines (e.g., TGFβ, IL-10) [16, 17]. Moreover, the mechanisms of immune tolerance may be further influenced by regional variables, including the variation of the immune cell populations within each tissue [18].

Immune tolerance has very significant implications in the context of critical illness. Critical illness associated with severe bacterial or viral infections (i.e., severe sepsis) or extensive trauma is characterized by an initial systemic pro-inflammatory response and subsequent immune suppression during which the host becomes susceptible to otherwise non-pathogenic microorganisms and activation of latent infections. Tolerance in the setting of critical illness is demonstrated in many cells, including cells that promote innate (e.g., macrophages) and adaptive (e.g., dendritic and T cells) immune responses, which explains susceptibility to an array of secondary infections and reactivation of latent infections [19, 20]. These secondary infections, and flaring of latent infections, contribute significantly to morbidity and mortality in the ICU setting [20–22]. An emerging body of evidence further indicates that treatments directed at reversing manifestations of immune tolerance, particularly T cell “exhaustion” consequent to enhanced expression of programmed cell death (PD-1) and PD-ligand 1 (PD-L1) and the related suppression of monocyte responsiveness, as reflected by reduced TNFα production, may be of significant benefit in terms of preventing secondary infections in the context of critical illness [20–22]. In this regard, IL-7, which potentiates T cell functions, is shown to reverse immune suppression in the context of severe sepsis [23].

We hypothesized that a murine splenocyte model would provide novel insights into the mechanisms by which LPS and CpG DNA induced tolerance and cross-tolerance, and that this model would be useful for exploring the means by which immune tolerance could be reversed. As hypothesized, these studies provide novel insight into the pathogenesis of immune suppression in the setting of acute critical illness, and provide support for the use of IL-7 to modulate tolerance and cross-tolerance under these conditions.

## Materials and Methods

### Ethics Statement

This study was carried out in strict accordance with the recommendations in the Guide for the Care and Use of Laboratory Animals of the National Institutes of Health. The protocol was approved by The Ohio State University Institutional Laboratory Animal Care and Use Committee (Approval Number: 2012000000197A). All efforts were made to minimize suffering, and tissue harvest was carried out immediately after approved euthanasia (70% carbon dioxide asphyxiation and cervical dislocation).

### Reagents

Murine CpG-containing oligonucleotide, type A (CpGA, TLR-9 ligand) was obtained from InvivoGen (San Diego, CA). Lipopolysaccharide (LPS, from *E*. *coli* Serotype 0111:B4) was acquired from Alexis Biochemicals (Enzo Life Sciences, Inc.; Farmingdale, NY). The rat, anti-mouse, polyclonal programmed death receptor-1 (PD-1) blocking antibody (RMP1-14) and corresponding isotypic control (PD-1C, RTK2758) antibody were obtained from BioLegend (San Diego, CA). Recombinant human IL-7 was provided by Cytheris SA (Rockville, MD). The primary antibody to interleukin-1 receptor-associated kinase 3 (IRAK-M) was acquired from Santa Cruz Biotechnology, Inc. (Santa Cruz, CA) with the corresponding secondary antibody attained from Cell Signaling Technology, Inc. (Danvers, MA). Mouse splenocytes (see below) were cultured using RPMI 1640 media (Life Technologies; Grand Island, NY). Unless otherwise stated, all additional chemicals were obtained from Sigma-Aldrich Corp. (St. Louis, MO) using the best available grade.

### Mouse pDC Expansion and Splenocyte Preparation

Eight-to-ten-week old, adult, male, C57BL/6 mice (~25 g) (The Jackson Laboratory; Bar Harbor, ME) (n = 14) were employed for *in vivo* pDC expansion and the subsequent source for all splenocytes. Additional corresponding experiments, however, utilized pDC-expanded splenocytes obtained similarly from matching IRAK-M knockout (IRAK-M-/-) mice (n = 3). pDC expansion was carried out using melanoma cells [expressing murine (Flt3)] as previously described [24, 25]. Briefly, 4 × 10^6^ B16 melanoma cells (C57BL/6 background), transfected with mouse recombinant Flt3 ligand (Flt3L) cDNA using an MFG-retroviral vector, were suspended in sterile saline and injected subcutaneously in two sites over both flanks. Mice were euthanized when the tumor size reached ~1–1.5 cm in diameter (after 3–4 weeks), and the spleens were harvested. Elevated pDC populations following Flt3L-induced expansion were confirmed via flow cytometry following immunofluorescent staining of splenocytes with a mouse anti-plasmacytoid dendritic cell antigen 1 (mPDCA-1)-FITC (JF05-1C2.4.1, Miltenyi Biotec, Inc.; Auburn, CA) antibody [11].

In previous studies [11, 26], we found that splenocytes obtained from normal, healthy untreated mice have unpredictable responses to murine CpGA DNA, presumably related to varying immature dendritic cell populations, even in littermates. Use of Flt3 ligand (Flt3L) expanded the immature dendritic cell populations (particularly pDCs) within the spleen, such that we were able to achieve highly uniform responses to TLR-9 ligands. Given the high cost of Flt3L, injection of species-matched B16 melanoma cells transfected with Flt3L served as a safe and inexpensive way to expose the bone marrow to Flt3L and achieve the desired effect, nearly doubling their presence in the total cell population within the spleen from 1–2% to ~4% after a 2–3 week exposure during tumor growth. Taking great care to obtain the spleen in advance of any negative effects of the abdominal tumor, the end result of this was to provide a splenocyte population that responded strongly and consistently to minimal to moderate doses of CpGA DNA across all cell preparations, enabling investigation of TLR-9 mediated immune responses. Since it was our intent to provide a model of an intact *in vivo* immune cell population that demonstrated tolerance/cross-tolerance to CpGA DNA, it became necessary to first establish one that would respond vigorously and consistently to CpGA DNA upon initial treatment.

Single cell splenocyte suspensions were prepared following mechanical disaggregation of the spleen tissue and gently passing the released cells and tissue fragments through a 70 μm nylon cell strainer. Following erythrocyte lysis, cells were washed and then suspended in RPMI 1640 (supplemented with 25 mM HEPES, 2 mM L-glutamine, 50 U/ml penicillin, 50 μg/ml streptomycin, and 10% FBS) (Life Technologies). Cells were then cultured in 48-well plates at a concentration of 1 × 10^6^/ml in the above media with only 2% FBS at 37°C in a 5% CO_2_-humidified atmosphere. After ~1 hour, desired concentrations of murine CpGA DNA (1 μM), LPS (10 ng/ml) or volume-matched vehicle (PBS) were then added to the medium. The cell supernatants were then carefully collected (minimizing cell disruption) at ~24 hours post-treatment and stored at -80°C for later analyses. The adherent cells were gently washed with pre-warmed PBS (~100 μl) and then cultured again as stated above in supplemented RPMI with 2% FBS. Following an additional hour for equilibration, subsequent additions of CpGA DNA, LPS or PBS were added to the medium as before providing matching or all cross-treatment combinations. All pre-incubations with PD-1 or PD-1C blocking antibodies (5 μg/ml) or recombinant human IL-7 (25 ng/ml) were made as a one-time dose 30 minutes prior to the aforementioned subsequent 24-hour ligand additions. Once again, cell supernatants were carefully collected after an additional 24 hours post-treatment and stored as before. Similar experiments involving successive matching and cross-treatment combinations of CpGA DNA, LPS or PBS, as described above, with or without IL-7 pre-treatment were carried out in Flt3L-expanded mouse splenocytes isolated from matching IRAK-M knockout (IRAK-M-/-) mice wherein IRAK-M depletion was confirmed as detailed previously [27, 28].

### Flow Cytometry Analysis

Following the treatment combinations identified above over 48 hours, splenocytes were collected and washed twice in cold FACS buffer. Cells were evaluated by three-color flow cytometry using a combination of the following directly conjugated antibodies (clones): PD-1 (CD279) (J43) or PD-L1 (CD274) (MIH5), CD11b (M1/70), CD11c (HL3), CD3 (17A2), CD4 (GK1.5), CD8a (53–6.7) and CD19 (1D3) (BD Biosciences; San Jose, CA), and PDCA-1 (JF05-1C2.4.1) (Miltenyi Biotec, Inc.; San Diego, CA) or the appropriate directly conjugated isotype controls for 20 minutes in the dark at 4°C. Cells were then washed twice in FACS buffer and re-suspended in 300 μl of FACS buffer for flow cytometry analysis. All flow samples were processed on an Accuri C6 flow cytometer, and the results analyzed using Accuri C6 software (BD Biosciences).

### Cytokine Measurements

Mouse splenocyte supernatants, collected at ~24 hours subsequent to each treatment series (i.e., at both 24 and 48 hours post-initial treatment), were analyzed for their IFNγ, TNFα (eBioscience, Inc.; San Diego, CA) and IL-10 (BD Biosciences; San Diego, CA) concentrations by ELISA according to the manufacturer’s recommendations. Cytokine ELISA sensitivities were 15, 8 and 16 pg/ml respectively.

### IRAK-M Signaling in Mouse Splenocytes

In some instances, mouse splenocytes were cultured and treated similarly in 6-well plates to provide enough cells for later Western blot analyses. Cells receiving matching treatments over the first and second 24-hour periods with and without intervening IL-7 pre-treatment at 24 hours post-treatment were collected after the entire 48-hour experiment and processed with added protease inhibitors for Western blot analyses according to standard techniques. IRAK-M expression in mouse splenocytes was then determined by employing a rat, anti-mouse polyclonal primary antibody (1:1000). Given that both β-actin and GAPDH protein expressions were observed to increase with treatment, protein band densities corresponding to IRAK-M expression were normalized to protein load established by parallel use of silver-stained gels (Pierce silver stain kit, Thermo Fisher Scientific; Rockford, IL) [29] and then compared to corresponding bands from untreated cells.

### Statistical Analyses

The data was derived from independent experiments, as designated in the figure legends, and was expressed as mean SD, and statistical significance was based upon a value of *p* ≤ 0.05. SigmaPlot 12.0 and SYSTAT 13.0 software were used to plot the data and carry out the statistical analyses, respectively. Splenocyte cytokine release in response to various treatment combinations over time (devised to identify tolerance/cross-tolerance) was compared to that from untreated controls (Vehicle, Vehicle) using the Kruskal-Wallis test. Where appropriate, *post hoc* analyses between group rank means were performed using Dunn’s test. Additional comparisons between experimental groups and corresponding untreated controls involving pre-treatment inhibition studies (i.e., PD-1 blocking antibody, IL-7) designed to potentially influence tolerance/cross-tolerance, use of IRAK-M-/- splenocytes intended to evaluate the role of IRAK-M in tolerance/cross-tolerance, and flow cytometry analyses of PD-1 and PD-L1 expression and Western blot evaluation of IRAK-M expression following splenocyte activation were performed using the Wilcoxon Rank-Sum test.

## Results

### Post-Treatment Characterization of Splenocyte Populations Demonstrated Similar Cell Viability

Knowing that cell death occurs in splenocyte populations over time which can be affected by factors such as cell stimulation and given our desired focus on the post-treatment period wherein tolerance/cross-tolerance can occur, we evaluated our experimental preparations for their total protein concentration as a reflection of the total splenocyte viability after the first 24-hour treatment period and following the removal of all non-viable cells by washing (see Methods). Thus, the total protein concentration was reflective of the total viable splenocyte population. The total protein concentration post-treatment was 112.8 ± 2.9 following CpGA treatment and 110.3 ± 3.6 following LPS treatment as a percentage of that for the control (vehicle-treated) group (100.0 ± 5.7) (NS). IL-7 pre-treatment yielded similar findings when compared to treatment alone: 118.2 ± 2.9 for IL-7 + CpGA (*p* < 0.05), and 107.2 ± 3.2 for IL-7 + LPS (NS). The small but statistically significant increase in total splenocyte protein concentrations following CpGA treatment in the presence of IL-7 may be related to its known effects on immune cell turnover, including reduced immune cell apoptosis and enhanced proliferation [30, 31]. These findings demonstrated that the total splenocyte viability was very similar among the groups allowing for comparative analyses during the post-treatment-induced tolerance/cross-tolerance period.

### LPS Tolerance Selectively Related to Pro-Inflammatory Cytokines

**Fig 1:**
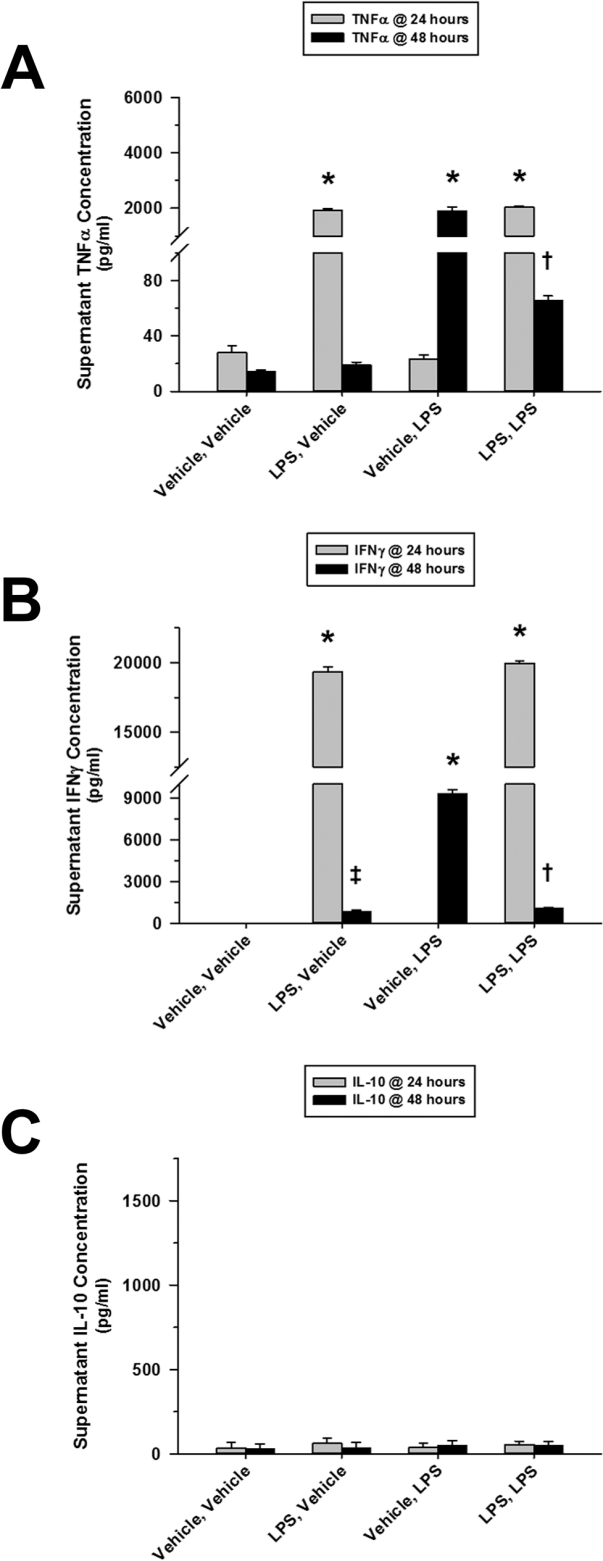
Repeated exposure to TLR-4 ligand, LPS, induces tolerance to splenocyte pro-inflammatory cytokine release. The presented data was derived from at least 4 independent experiments, each in triplicate. (A) LPS (10 ng/ml) induced significant TNFα release from cultured Flt3L-expanded mouse splenocytes (1 × 10^6^ cells/ml) 24 hours post-treatment. In response to a subsequent LPS treatment, TNFα release was dramatically reduced 24 hours later. (B) Similarly, significant splenocyte IFNγ release was observed 24 hours following LPS treatment. IFNγ release was markedly decreased 24 hours after a successive LPS treatment. This release, however, was very similar to the sustained IFNγ release observed over the subsequent 24-hour period in the absence of follow-up treatment (LPS, Vehicle). (C) Splenocytes failed to demonstrate any IL-10 release in response to LPS treatment [*p < 0.01, relative to time-matched untreated (Vehicle, Vehicle) controls; ^†^ *p* < 0.01, compared to the associated LPS treatment group at 24 hours (LPS, LPS) and the time-matched LPS treatment alone group (Vehicle, LPS), and ^‡^ *p* < 0.01, relative to the time-matched untreated (Vehicle, Vehicle) control and the associated LPS treatment group at 24 hours (LPS, Vehicle)].

LPS treatment of cultured splenocytes resulted in near complete tolerance to subsequent LPS treatment 24 hours later, as reflected by dramatically suppressed TNFα release (Fig 1A). Notably, LPS induced sustained IFNγ release which extended to 48 hours in subsequently untreated cells, and this sustained IFNγ release was observed to be nearly identical to that following successive LPS treatment-induced tolerance (Fig 1B). Finally, LPS did not induce any IL-10 release (Fig 1C). As such, IL-10 cannot be implicated in the induction of tolerance to LPS.

### CpG DNA Tolerance Non-Selectively Influenced Both Pro- and Anti-Inflammatory Cytokines

**Fig 2:**
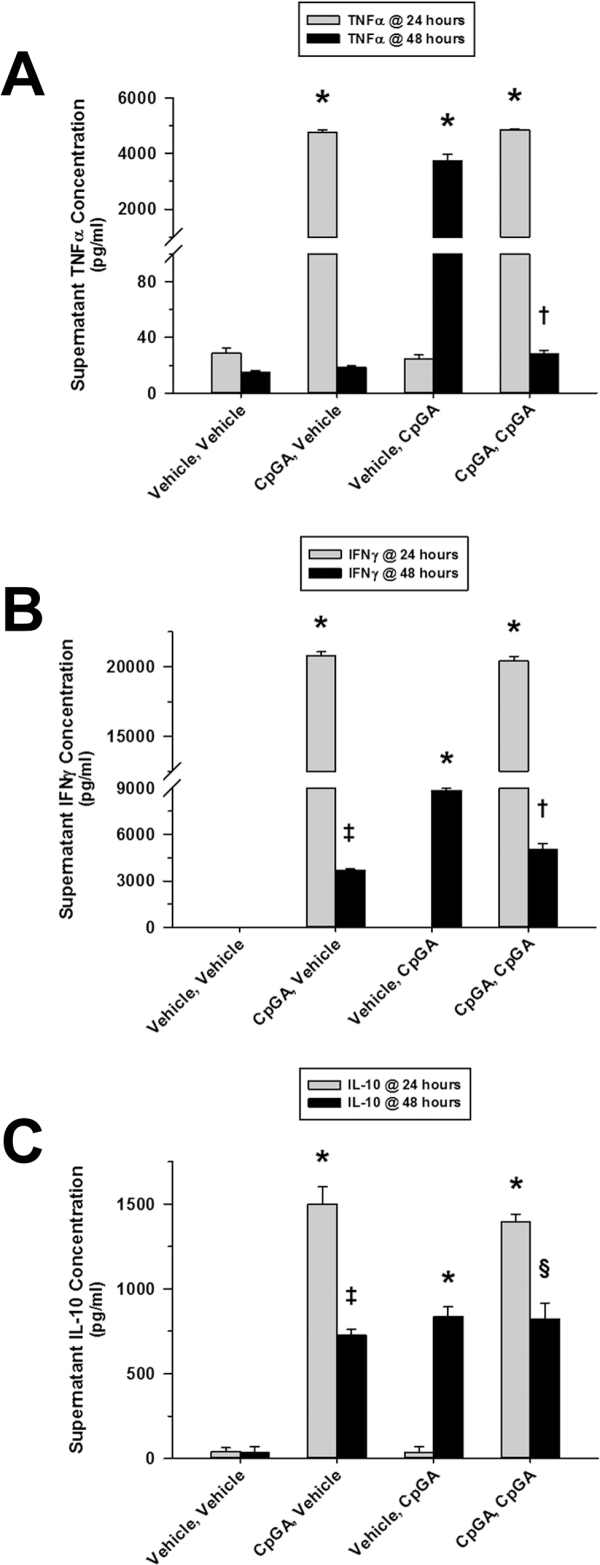
Tolerance to splenocyte cytokine release results from repeated exposures to TLR-9 ligand, CpGA DNA. The presented data was derived from at least 4 independent experiments, each in triplicate. (A) 24 hours post-treatment, CpGA DNA (1 μM) significantly induced Flt3L-expanded mouse splenocyte (1 × 10^6^ cells/ml) TNFα release. This effect was notably attenuated 24 hours after subsequent treatment with CpGA DNA. (B) Likewise, splenocyte exposure to CpGA DNA yielded marked release of IFNγ by 24 hours post-treatment. Successive treatment with CpGA DNA resulted in a significant reduction in IFNγ release which was observed to be comparable to the sustained IFNγ release typically demonstrated during the subsequent 24 hours when untreated (CpGA, Vehicle). (C) Splenocyte IL-10 release in response to repeated treatments with CpGA DNA yielded a pattern similar to that observed with IFNγ release [*p < 0.01, compared to time-matched untreated (Vehicle, Vehicle) controls; ^†^ *p* < 0.05, relative to the associated CpGA treatment group at 24 hours (CpGA, CpGA) and the time-matched CpGA treatment alone group (Vehicle, CpGA); ^‡^ *p* < 0.01, compared to the time-matched untreated (Vehicle, Vehicle) control and the associated CpGA treatment group at 24 hours (CpGA, Vehicle), and ^§^ *p* < 0.01, relative to the associated CpGA treatment group at 24 hours (CpGA, CpGA) only].

CpGA DNA treatment of cultured splenocytes resulted in tolerance to subsequent CpGA DNA treatment. Like LPS tolerance, CpGA DNA treatment strongly suppressed TNFα release to a subsequent CpGA DNA treatment (Fig 2A). Moreover, IFNγ release following CpGA DNA treatment, as with LPS, was sustained for at least 48 hours and was observed to be quite similar to that following successive CpGA DNA treatment-induced tolerance (Fig 2B). Unlike the situation with LPS treatment but as was observed with IFNγ release in this setting, IL-10 release following CpGA DNA treatment was sustained for at least 48 hours which was not different from that resulting from a tolerance-induced secondary exposure to CpGA DNA (Fig 2C).

### LPS and CpG DNA Cross-Tolerance Were Not the Same

**Fig 3:**
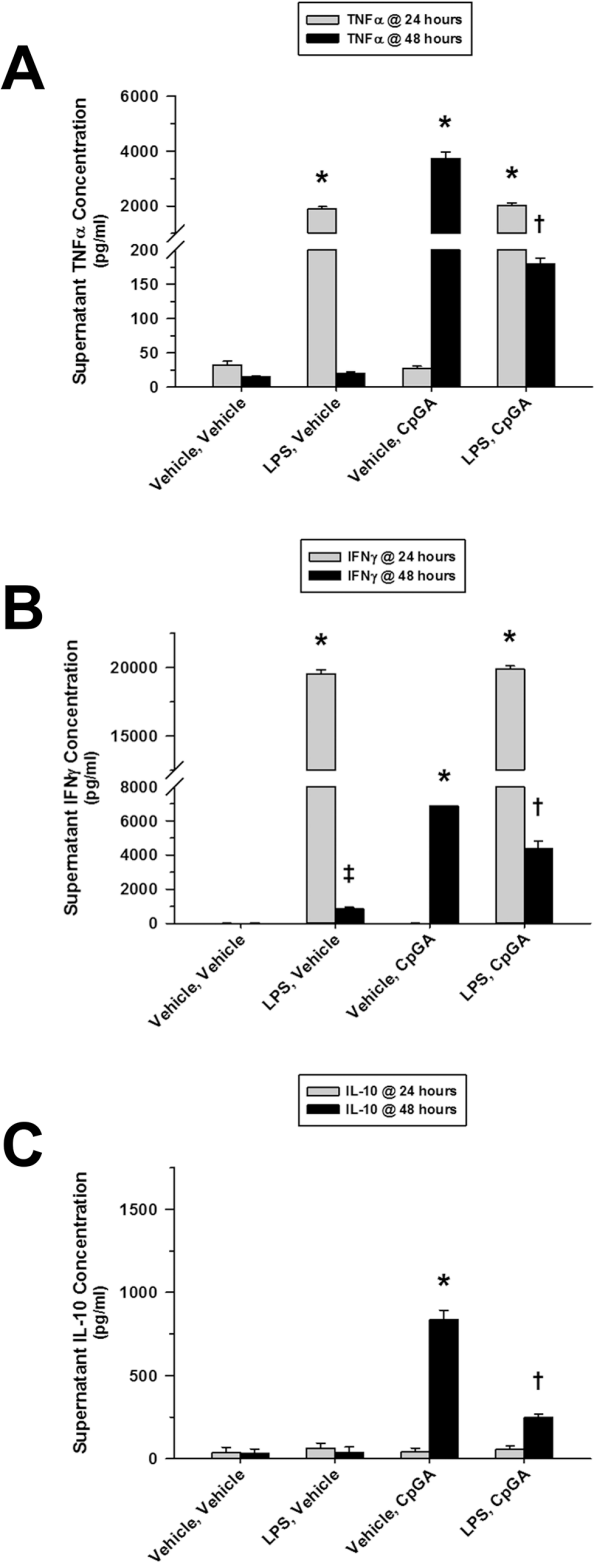
Cross-tolerance to splenocyte cytokine release results from Sequential exposures to LPS followed by CpGA DNA. The presented data was derived from at least 4 independent experiments, each in triplicate. (A) Flt3L-expanded splenocyte (1 × 10^6^ cells/ml) TNFα release was dramatically diminished 24 hours following treatment with CpGA DNA (1 μM) after a prior 24-hour exposure to LPS (10 ng/ml). (B) Similarly, following a previous 24-hour exposure to LPS, splenocyte IFNγ release was significantly suppressed 24 hours after treatment with CpGA DNA. (C) Consistently, splenocyte IL-10 release demonstrated the same pattern of cross-tolerance in response to CpGA DNA at 24 hours post-treatment after an earlier 24-hour exposure to LPS [*p < 0.01, compared to time-matched untreated (Vehicle, Vehicle) controls; ^†^ *p* < 0.05, relative to the time-matched CpGA treatment alone group (Vehicle, CpGA), and ^‡^ *p* < 0.01, compared to the time-matched untreated (Vehicle, Vehicle) control and the associated LPS treatment group at 24 hours (LPS, Vehicle)].

LPS treatment of cultured splenocytes resulted in cross-tolerance to subsequent CpGA DNA treatment. CpGA DNA treatment 24 hours after LPS exposure resulted in significant suppression of both TNFα (Fig 3A) and IFNγ (Fig 3B) release, which was more dramatic for TNFα. LPS exposure also attenuated IL-10 release in response to subsequent CpGA DNA treatment (Fig 3C).

**Fig 4:**
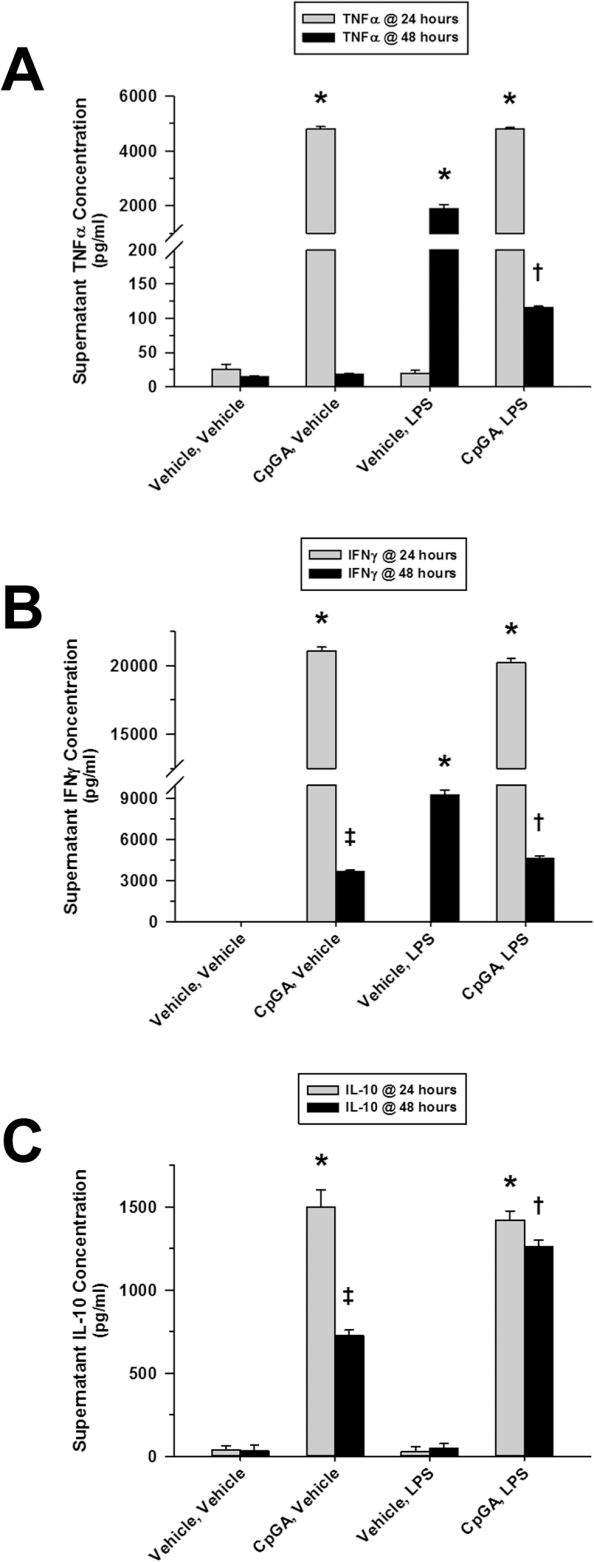
Sequential exposures to CpGA DNA followed by LPS lead to cross-tolerance to splenocyte cytokine release. The presented data was derived from at least 4 independent experiments, each in triplicate. (A) Following a prior 24-hour exposure to CpGA DNA (1 μM), TNFα release from Flt3L-expanded mouse splenocytes (1 × 10^6^ cells/ml) was significantly reduced 24 hours after subsequent treatment with LPS (10 ng/ml). (B) Likewise, splenocyte IFNγ release was markedly diminished 24 hours after treatment with LPS following a previous 24-hour exposure to CpGA DNA. (C) Surprisingly, splenocyte IL-10 release was significantly and notably augmented in response to LPS at 24 hours post-treatment after an earlier 24-hour exposure to CpGA DNA [*p < 0.01, relative to time-matched untreated (Vehicle, Vehicle) controls; ^†^ *p* < 0.05, compared to the time-matched LPS treatment alone group (Vehicle, LPS), and ^‡^ *p* < 0.01, relative to the time-matched untreated (Vehicle, Vehicle) control and the associated CpGA treatment group at 24 hours (CpGA, Vehicle)].

CpGA DNA treatment of cultured splenocytes resulted in selective cross-tolerance to subsequent LPS treatment for TNFα and IFNγ, but not IL-10. As was the case for cross-tolerance induced by LPS, initial exposure to CpGA DNA resulted in suppression of TNFα (Fig 4A) and IFNγ (Fig 4B) following subsequent treatment with LPS. However, it was interesting to note that CpGA DNA treatment actually primed splenocytes for the production of IL-10 following subsequent exposure to LPS (Fig 4C).

### PD-1/PD-L1 Did Not Influence Immune Tolerance within 48 hours of LPS or CpG DNA Treatment

**Fig 5:**
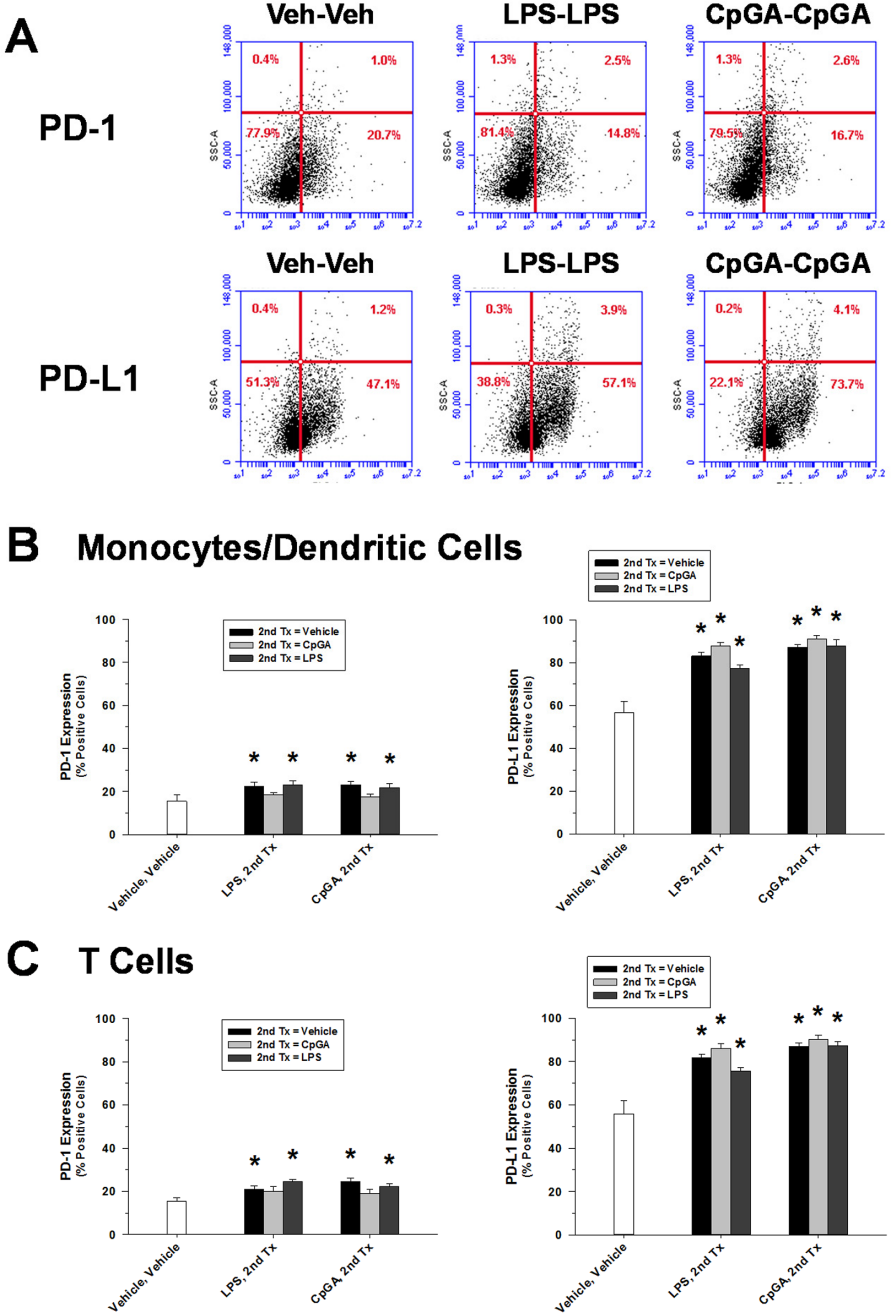
Splenocyte membrane expressions of both PD-1 and PD-L1 increase following TLR-4/TLR-9 stimulation. The presented data was derived from at least 3 independent experiments. Data represents the percentage of grouped splenocyte populations demonstrating both PD-1 and PD-L1 membrane expression by flow cytometry analysis 48 hours after successive 24-hour exposures to the ligand treatment combinations detailed on the X-axis. (A) Representative flow cytometry scatter plots demonstrating that the percentage of both PD-1- and PD-L1-expressing splenocytes increased after successive stimulation with either LPS or CpGA DNA compared to Vehicle, Vehicle (Veh, Veh)-treated controls. (B) Both PD-1 (left) and PD-L1 (right) membrane expression levels were significantly increased in monocytes and dendritic cells after either LPS or CpGA DNA exposure. (C) Likewise, following either LPS or CpGA DNA treatment, T cells demonstrated elevated membrane expression levels of both PD-1 (left) and PD-L1 (right) identical to those observed in the monocytes/dendritic cells [PD-1 expression: *p < 0.05, compared to the untreated (Vehicle, Vehicle) control group (white bar); PD-L1 expression: *p < 0.05, relative to the untreated (Vehicle, Vehicle) control group (white bar). Following an initial given treatment (i.e., LPS or CpGA DNA), there was no statistical difference among the 3 secondary treatment groups (shaded bars) for either PD-1 (left) or PD-L1 (right).].

**Fig 6:**
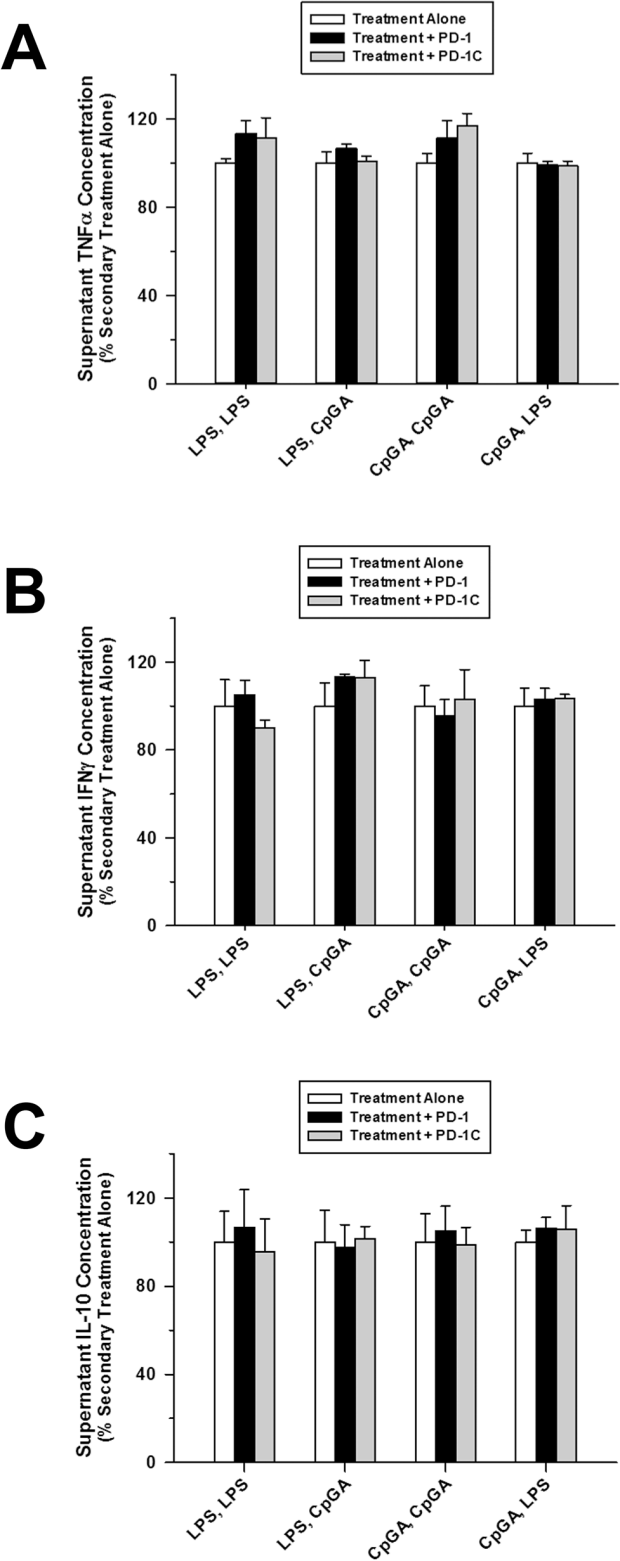
Tolerance and cross-tolerance to splenocyte cytokine release is unaffected by blocking PD-1. The presented data was derived from at least 3 independent experiments, each in triplicate. Data represents splenocyte cytokine release 48 hours after successive 24-hour exposures to the ligand treatment combinations detailed on the X-axis. (A) Pre-treatment (30 minutes) of Flt3L-expanded splenocytes (1 × 10^6^ cells/ml) with an mouse anti-PD-1 blocking antibody (or corresponding isotypic control antibody, PD-1C) (5 μg/ml) prior to the secondary 24-hour ligand treatment had no effect upon resultant TNFα release regardless of the treatment combination employed. (B) Similarly, anti-PD-1 pre-treatment did not alter resultant splenocyte IFNγ release for any of the treatment combinations used. (C) Consistently, resultant splenocyte IL-10 release was unaffected for all treatment combinations by anti-PD-1 pre-treatment.

PD-1/PD-L1 expression was promoted in both monocyte/macrophage lineage cells and T cell lineage cells (Fig 5B and 5C, respectively) in response to either LPS or CpGA DNA. Notably, the percentage of cells expressing PD-L1 was nearly 4 times greater than for PD-1-expressing cells (Fig 5). Of note in this regard, splenocytes are a heterogeneous population of cells, and the slight variability in the side scattered light (SSC) is considered to be due to specific populations responding to the activation stimuli (and resultant enhanced expression of PD-1/PD-L1). Activation of cells, particularly myeloid cells, can change the internal composition/granularity/complexity within these cells and/or induce some degree of proliferation which will result in an increased SSC and signal variability (see S1 Fig). Despite the observed induction of PD-1, anti-PD-1 pre-treatment had no effect upon tolerance or cross-tolerance in response to treatment with either CpGA DNA or LPS (Fig 6).

### IL-7 Attenuated Tolerance and Cross-Tolerance to LPS and CpG DNA

**Fig 7:**
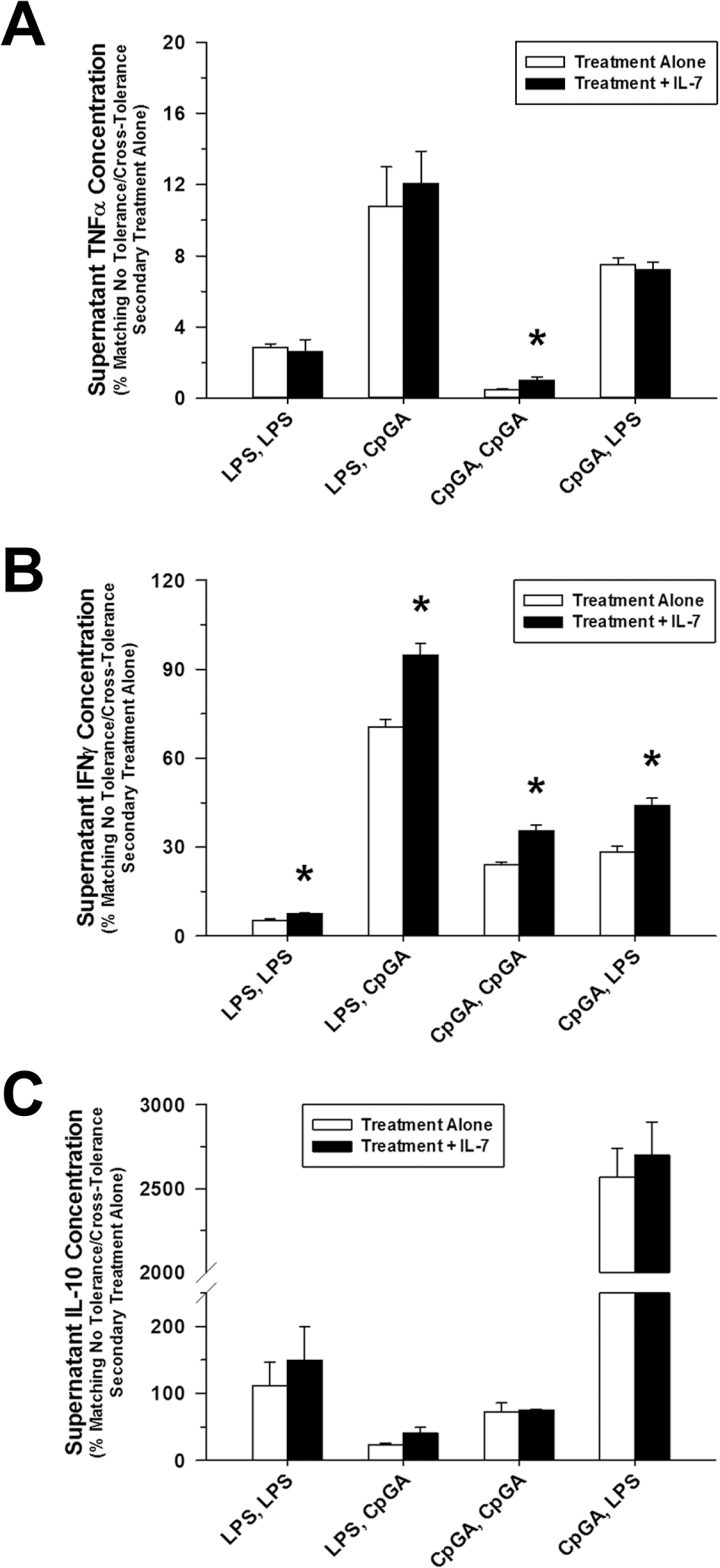
IL-7 pre-treatment restores responsiveness to subsequent ligand exposures attenuating tolerance and cross-tolerance. The presented data was derived from at least 3 independent experiments, each in triplicate. Data represents splenocyte cytokine release (as a percent of the matching no tolerance/cross-tolerance [i.e., (Vehicle, LPS) or (Vehicle, CpGA), respectively] secondary ligand exposure alone) 48 hours after successive 24-hour exposures to the ligand treatment combinations detailed on the X-axis. (A) Following a 24-hour exposure to CpGA DNA (1 μM), pre-treatment (30 minutes) of Flt3L-expanded mouse splenocytes (1 × 10^6^ cells/ml) with human recombinant IL-7 (25 ng/ml) prior to the subsequent ligand treatment significantly improved responsiveness to CpGA DNA-induced TNFα release 24 hours post-treatment. (B) Regardless of the treatment combination employed, tolerance and cross-tolerance to splenocyte IFNγ release in response to the secondary ligand exposure was attenuated by IL-7 pre-treatment. (C) Pre-treatment with IL-7 had no significant effect upon splenocyte IL-10 release resulting from the secondary ligand exposure [*p < 0.05, compared to the matching secondary treatment alone (white bar)].

Although dramatic tolerance/cross-tolerance had been observed in most settings for all cytokines evaluated, IL-7 pre-treatment was observed to particularly improve responsiveness in terms of TNFα release only in the setting of successive CpGA DNA treatments, though significant tolerance remained (Fig 7A). However, IL-7 pre-treatment markedly suppressed tolerance and cross-tolerance to both LPS and CpG DNA in terms of IFNγ responses (Fig 7B), which was in keeping with the established T cell stimulatory effects of IL-7 [21]. Notably, IL-7 did not significantly reverse tolerance or cross-tolerance in terms of IL-10 release (Fig 7C), the implications of which are discussed below.

### IRAK-M Mediated Tolerance and Cross-Tolerance to LPS and CpG DNA

**Fig 8:**
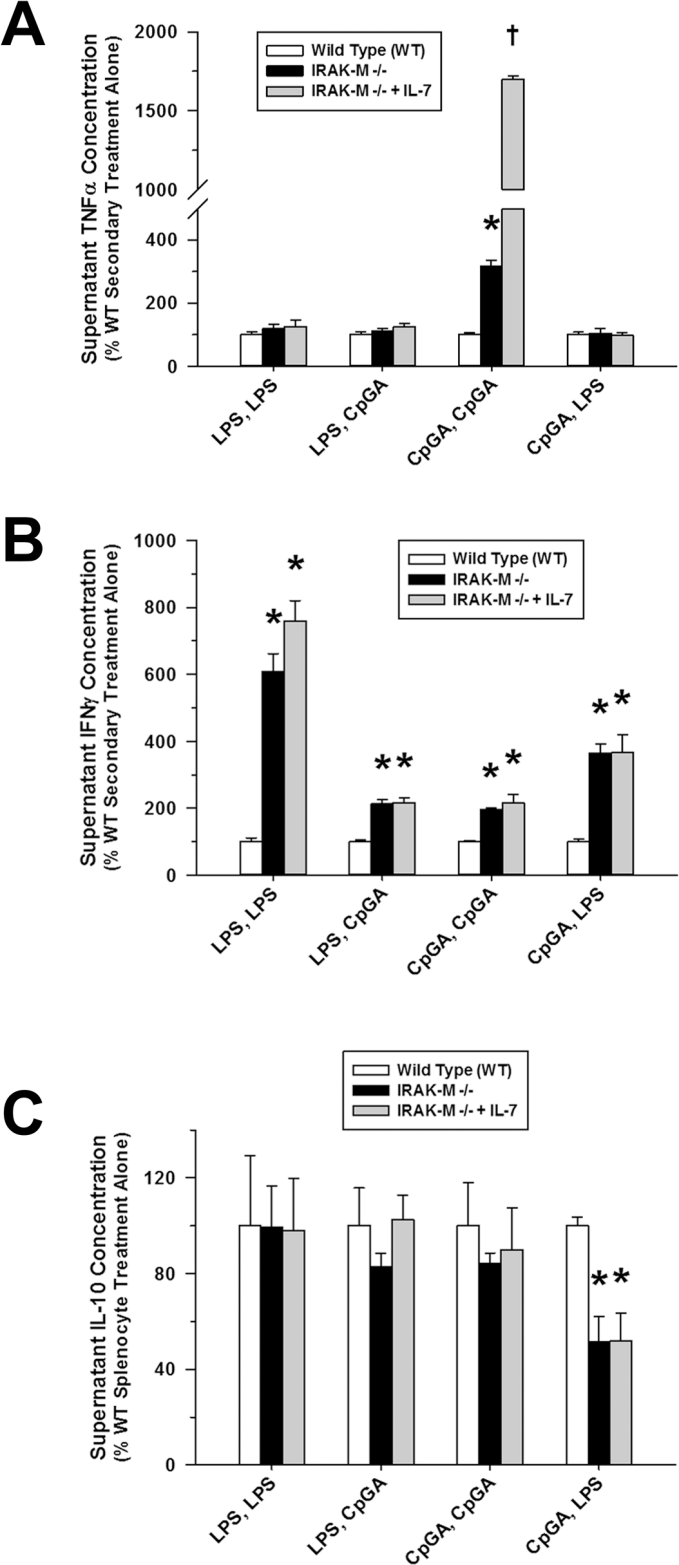
IRAK-M-/- splenocytes demonstrate diminished tolerance and cross-tolerance through improved responsiveness to subsequent ligand exposures. The presented data was derived from at least 3 independent experiments, each in triplicate. Data represents splenocyte cytokine release [as a percent of secondary ligand exposure in matching wild-type (WT) splenocytes] 48 hours after successive 24-hour exposures to the ligand treatment combinations detailed on the X-axis. (A) Following a 24-hour exposure to CpGA DNA (1 μM), Flt3L-expanded mouse IRAK-M-/- splenocytes (1 × 10^6^ cells/ml) showed significantly improved responsiveness to subsequent CpGA DNA-induced TNFα release at 24 hours post-treatment which was further augmented by IL-7 (25 ng/ml) pre-treatment (30 minutes). (B) Regardless of the treatment combination employed, tolerance and cross-tolerance to IRAK-M-/- splenocyte IFNγ release in response to the secondary ligand exposure was significantly attenuated. This observation was unaffected by IL-7 pre-treatment. (C) IRAK-M-/- splenocytes demonstrated no enhancement in IL-10 responsiveness to the secondary ligand exposure regardless of IL-7 pre-treatment [*p < 0.01, relative to the matching wild-type secondary treatment (white bar), and ^†^ *p* < 0.01, compared to the matching CpGA DNA secondary treatment in the absence of IL-7 (black bar)].

**Fig 9:**
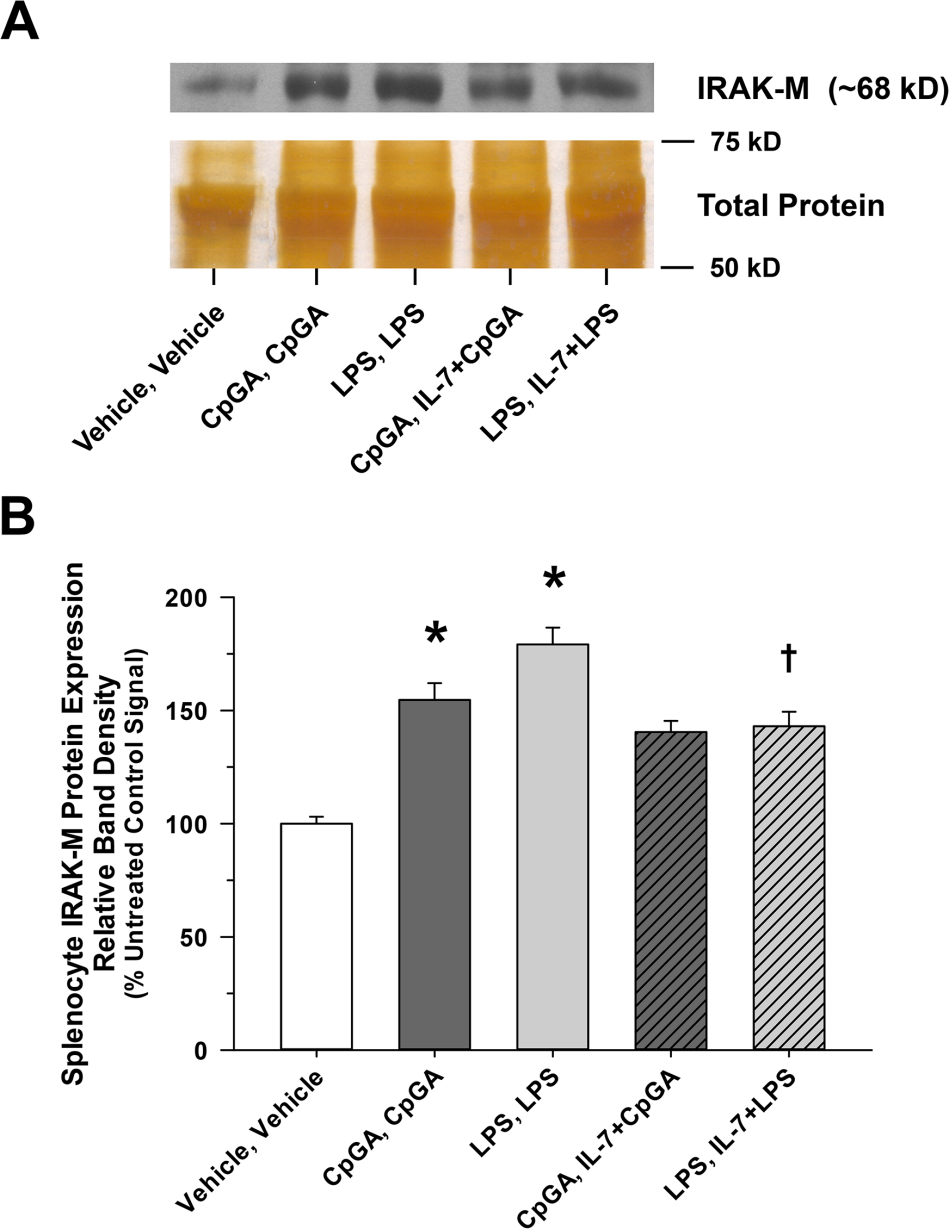
Elevated splenocyte IRAK-M expression in response to repeated TLR-4 or TLR-9 ligand exposures is suppressed by IL-7 pre-treatment. The presented data is representative of at least 3 independent experiments. (A) Representative photomicrograph of a Western blot demonstrating the changes observed in IRAK-M expression 48 hours after successive 24-hour repeated ligand exposures (with or without a 30 minute IL-7 pre-treatment). (B) Relative band density of splenocyte IRAK-M expression was dramatically elevated for both successive treatment combinations and notably reduced with intervening IL-7 pre-treatment [*p < 0.05, relative to the untreated (Vehicle, Vehicle) control group, and ^†^ *p* < 0.05, compared to the corresponding LPS treatment combination in the absence of IL-7 (LPS, LPS)].

Given the putative role of IRAK-M, a natural negative regulator of TRAF6/IRAK-1 and downstream NF-κB signaling, in the development of tolerance to TLR stimuli, including TLR-4 and TLR-9 [27], we sought to determine whether IRAK-M expression was essential for the induction of immune tolerance in the splenocyte culture model. As illustrated in Fig 8, splenocytes derived from IRAK-M-/- mice released significantly more TNFα compared to matching wild-type splenocytes in response to repeated treatments with CpGA DNA (Fig 8A) and comparatively higher levels of IFNγ in response to any combination of LPS and CpGA DNA treatments (Fig 8B). Interestingly, IRAK-M-/- mice had suppressed IL-10 release following successive treatments with CpGA DNA and LPS (Fig 8C). We then sought to determine whether IL-7 influenced IRAK-M expression during tolerance to LPS and CpGA DNA in the splenocyte culture model. As demonstrated in Fig 9, IL-7 significantly reduced IRAK-M expression during LPS-induced tolerance but did not significantly affect IRAK-M expression during CpGA DNA-induced tolerance (though improving TNFα responsiveness to repeated CpGA DNA treatments, Fig 8A).

## Discussion

This study explores the mechanisms of tolerance and cross-tolerance in a splenocyte culture model that closely approximates the complex immune cell interactions *in vivo*. In an effort to more accurately model conditions observed in acutely ill patients (e.g., with severe bacterial or viral infections), this model was modified such that the initial doses of LPS and CpGA DNA used to induce tolerance were sufficient to produce an acute, intense immune response, such as occurs in the setting of severe acute illness [32, 33]. The profound tolerance and cross-tolerance observed in the splenocyte culture model is comparable to the results of previous studies conducted on isolated macrophage preparations [1, 34] and in humans treated with LPS [15] and is in keeping with tolerance observed in animal sepsis models [3] and in patients with severe sepsis and trauma [35–37]. These observations, and the mechanisms considered herein, are highly relevant and have important implications for the development of secondary infections, including nosocomial bacterial infections and reactivation of latent viral infections, in the context of critical illness [20–22].

As expected based upon studies performed on immortalized macrophage cell lines [1, 27] and in mice [38], high-dose LPS treatment of murine splenocytes promoted significant tolerance to subsequent LPS treatment and cross-tolerance to subsequent CpGA DNA treatment. Highly purified LPS, as was used in these experiments, is a selective TLR-4 agonist, a receptor that is essential for the host defense against Gram-negative bacterial infections [39, 40]. Moreover, tolerance and cross-tolerance induced by LPS non-selectively suppressed pro- and anti-inflammatory cytokine release, as previously reported following human endotoxemia [2, 4]. Compared to immortalized cell lines or single cell cultures, this model more closely replicates conditions of severe bacterial sepsis or trauma wherein the initial immune response is characteristically very intense (e.g., described as a “cytokine storm”) [32, 33]. In this regard, it is important to note that profound tolerance and cross-tolerance to high-dose LPS was established quickly (within 24 hours), as was previously reported in humans [41]. It follows that the stage is rapidly set for secondary bacterial, viral and fungal infections and reactivation of latent viruses, which contribute significantly to patient morbidity and mortality in critically ill patients [22].

Comparatively less is known about tolerance and cross-tolerance following CpG DNA exposure, which promotes both pro- and anti-inflammatory cytokine release via TLR-9 signaling. Like LPS, high-dose CpGA DNA exposure strongly induced tolerance to subsequent CpGA DNA or LPS treatment, particularly in terms of pro-inflammatory cytokine release. Unexpectedly, secondary exposure to LPS following CpGA DNA treatment led to the selective release of significant amounts of IL-10. To the extent that the splenocyte culture model reflects conditions *in vivo*, this finding suggests that a secondary Gram-negative bacterial infection following exposure to CpG DNA (e.g., DNA virus infection, or mitochondrial DNA release following trauma) could lead to further suppression of the immune response. This mechanism may contribute to the observed predisposition for bacterial super-infections following DNA viral infections [42, 43].

In terms of mechanisms by which immune tolerance is promoted within a mixed population of immune cells in the spleen, extracellular factors would seem to be the most plausible explanation. IL-10 is implicated as an important mediator of immune tolerance to LPS [44, 45]. IL-10 is also implicated in the development of secondary bacterial infections following viral infections [46] or trauma [47], conditions associated with TLR-9 activation [48–50]. However, our data did not support a primary role of IL-10 in the development of tolerance or cross-tolerance in the spleen in response to LPS or CpGA DNA. For instance, no IL-10 was produced in response to the initial dose of LPS after which profound tolerance and cross-tolerance was observed (Fig 1C) and had no correlation with TNFα or IFNγ responses to CpGA DNA (Fig 2). That said, it is interesting to note that initial CpGA DNA treatment primed the splenocytes for IL-10 release following subsequent LPS exposure (Fig 4C). This finding implies that sequential TLR-9 and TLR-4 stimulation are additive in terms of promoting immune suppression.

Programmed death receptor-1 (PD-1) is another well-characterized mediator of immune tolerance and is shown to suppress immune responses to LPS in the RAW264.7 macrophage cell line [51]. Thus, it is not unexpected that PD-1 and PD-L1 expressions were significantly promoted by treatment combinations of LPS or CpGA DNA with successive challenges of either vehicle, LPS or CpGA DNA (Fig 5). Of note, baseline PD-L1 expression was much higher in both monocyte and T cell lineage cells within the splenocyte preparation compared to that of PD-1. In contrast to immune suppression during established sepsis, wherein PD-1/PD-L1 interactions between antigen-presenting cells and T cells are shown to strongly influence immune hyporesponsiveness [52–54], anti-PD-1 antibody treatment did not influence LPS- or CpGA DNA-induced tolerance or cross-tolerance in the splenocyte model (Fig 6). Our findings are consistent with previous studies showing PD-1 to be expressed minimally on resting T cells compared to activated T cells wherein PD-1 is inducibly expressed and is functional within 24 hours with maximal expression occurring after 48 hours [55]. Since this study was limited to tolerance induced within 24 hours and given that expression levels were increasing significantly at that time, it remains plausible that PD-1/PD-L1 interactions could contribute to tolerance and cross-tolerance, especially relating to T cell functions (e.g., IFNγ release), that typically occurs several days after TLR-4 or TLR-9 activation.

In keeping with its documented capacity to promote dendritic cell-mediated T cell activation [56] and restoration of T cell responses (e.g., IFNγ) in the setting of sepsis [30], IL-7 treatment selectively restored IFNγ release in response to tolerance and cross-tolerance following treatment with high doses of either LPS or CpGA DNA (Fig 7B). T cells are particularly responsive to IL-7 given that their basal NF-κB activity plays an important role in enhancing their sensitivity to IL-7, even in the absence of antigen encounter, thus promoting T cell viability and responsiveness [57]. Restoration of T cell dysfunction, particularly IFNγ production, is shown to be critical for the resolution of infections during critical illness, as reflected by recent laboratory [53] and human studies [4, 58]. IL-7 also partially restored responsiveness in the form of TNFα release to CpGA DNA, a selective TLR-9 agonist (Fig 7A). This data indicates that the function of TLR-9-responsive splenocytes, particularly dendritic cells, is augmented by IL-7 in the setting of CpG DNA-mediated tolerance. With regard to restoring immune responsiveness in the setting of tolerance and cross-tolerance, it is important to note that IL-7 does not promote IL-10 release, which would potentially counterbalance the restoration of pro-inflammatory cytokines, including IFNγ and TNFα.

The likely role of IRAK-M, a putative mediator of LPS-induced tolerance [13, 59], in the induction of tolerance and the reversal thereof by IL-7 was supported to some degree by our data. IRAK-M is an endogenous negative regulator of TRAF6/IRAK-1 and downstream NF-κB signaling that is induced by LPS [27, 38] and by CpG DNA [60]. As demonstrated in Fig 8, splenocytes from IRAK-M-/- mice exhibited significantly less tolerance and cross-tolerance for certain immune responses, particularly TNFα release following successive treatments with TLR-9 agonist, CpGA DNA, and IFNγ (presumably T cell-mediated) release in response to any serial treatment combination of TLR-4 and TLR-9 agonists. The role of IRAK-M during tolerance was further supported by the observed induction of IRAK-M protein expression in the splenocyte model following either LPS or CpGA DNA treatment (Fig 9). IL-7 significantly but incompletely suppressed IRAK-M expression following LPS treatment, but had no significant effect following CpGA DNA treatment (Fig 9B). It is notable that other intracellular mechanisms are proposed to regulate tolerance and cross-tolerance following TLR stimulation, including endogenous negative regulators (e.g., SHIP-1, A20) [38], and microRNA-mediated inhibition of components of the MyD88-dependent signaling pathway [61, 62]. In fact, Zhou et al recently demonstrated that through its association with MyD88 and IRAK-4, IRAK-M mediates TLR-induced MEKK3-dependent secondary activation of NF-κB to produce the aforementioned inhibitory regulators, including SOCS1 and IκBα, as well as exacting an inhibitory effect upon the translational control of microRNA-mediated cytokine production and resultant inflammation [14]. These mechanisms appear to be most relevant to the suppression of monocyte/macrophage cell lines, which could account for the suppression of TNFα [61, 62] and perhaps IFNγ [41] in the current study. With respect to TLR-4 tolerance in particular, SHIP is known to contribute to LPS tolerance through the inhibition of NF-κB [63], and SOCS-1 regulates LPS responses through inhibition of JAK-STAT pathways [64]. Additional experiments are required to clarify these and alternate mechanisms by which tolerance and cross-tolerance in macrophage/dendritic cell lineages (e.g., blunted TNFα responses) and T cells (e.g., reduced IFNγ and IL-10 responses) occur in the setting of complex immune cell interactions, such as modeled herein and as occurs *in vivo* during critical illness.

Any attempt to model the immune response can be criticized; however, the *ex vivo* splenocyte culture model is considered to be a useful surrogate for complex *in vivo* immune responses in the setting of bacterial [65–67] and viral infections [68, 69], and “sterile” immune responses to endogenous danger signals [11, 26]. The complex interaction among the various immune cells and the changes in immune cell function following stimulation in the splenocyte model recapitulates the dynamic immune cell interactions and related disease mechanisms *in vivo*. The tradeoff of a complex model is the inability to clearly define mechanisms, such as the source of a given cytokine, or the immune cell that is responding to a specific treatment (e.g., IL-7). That said, we can glean mechanisms based upon the known functions of the cells that are present in the modified (Flt3-expanded) splenocyte preparations (e.g., immature dendritic cells primarily respond to TLR-9 agonists and are unresponsive to TLR-4 in splenocyte cultures [70], T cells are the primary source of IFNγ, and dendritic cells and T cells produce IL-10 [71]) and the treatments (e.g., IL-7 promotes dendritic cell and T cell activation [20]; whereas, PD-1/PD-L1 regulates T cell activation by antigen-presenting cells [52–54]). Admittedly, the immune response is complex and involves multiple cells and many intercellular interactions. In particular, NK cells are important participants in the immune response, particularly as a source of IFNγ, respond to both TLR-4 and TLR-9 agonists, and are rendered tolerant in response to activation by TLR agonists in the spleen. Recent studies indicate that regulatory T cells are critical for NK cell tolerance in the spleen under these conditions [72]. The details of these complex interactions are beyond the scope of the present study. However, we postulate that the splenocyte model more accurately replicates *in vivo* immune responses compared to reductionist models using a single immortalized or isolated cell line. Moreover, we can infer that IL-7 selectively restored the functions of TLR-9-responsive immature dendritic cells [e.g., to produce TNFα (Fig 7A)] and T cells (and consequently NK cells) [e.g., to produce IFNγ (Fig 7B)], and this effect may be related, in part, to changes in IRAK-M expression (Fig 9). Since IL-7 did not completely reverse tolerance or cross-tolerance, it is apparent that further studies are needed to consider other mechanisms, and related treatments, contributing to tolerance and cross-tolerance in response to acute TLR-4 or TLR-9 stimulation.

In summary, the profiles of tolerance and cross-tolerance following treatments with LPS or CpG DNA (TLR-4 and TLR-9 ligands, respectively) in a murine *ex vivo* splenocyte model are distinct. Tolerance and cross-tolerance to LPS was shown to be associated with dramatic and selective suppression of pro-inflammatory cytokines (TNFα, IFNγ); whereas, tolerance to CpGA DNA affected both pro- (TNFα, IFNγ) and anti-inflammatory (IL-10) cytokines. Additionally, CpG DNA was shown to prime the splenocytes to promote LPS-induced IL-10 release. The mechanisms of early immune tolerance in this model were unrelated to IL-10 or PD-1/PD-L1, and immune tolerance, particularly IFNγ suppression, was significantly reversed following treatment with IL-7. Furthermore, IL-7 did not promote IL-10 release. Together, these data demonstrate that IL-7 significantly reverses tolerance and cross-tolerance induced by LPS or CpG DNA, and to our knowledge, this is the first study to implicate IRAK-M as a target of IL-7-mediated activity. Thus, the murine splenocyte model, which closely replicates complex intercellular interactions *in vivo*, is shown to be useful for examining the mechanisms of tolerance and cross-tolerance consequent to exposure to bacterial or host danger signals, and for developing related treatment strategies, in the context of critical illness.

## Supporting Information

S1 Checklist The ARRIVE Guidelines Checklist provides direction as to where information related to the use of animals in the present study is located within the manuscript. (PDF)

S1 Fig Post-experimental analyses of the side-scattered light (SSC) demonstrated minimal variability among the experimental groups following flow cytometry evaluation of treatment-induced increases in splenocyte PD-1/PD-L1 expression. (A) Overlays of the various treatment groups demonstrated only minimal changes between Vehicle (Veh)/Non-treated (NT) versus treated groups. (B) Histogram of the mean SSC showing comparable SSC values for the NT-NT/Veh-Veh groups with an expected increase observed for the treated groups (LPS or CpG) typical of activated cell populations. (C) Detailed histogram statistics of the SSC signal confirmed similar SSC detection patterns in the NT-NT/Veh-Veh groups with slight increases in the mean and percentage of coefficient of variation of the SSC signal in the treated groups (LPS-LPS or CpGA-CpGA) consistent with the morphological changes expected with cell activation following treatment with pathogen-associated molecular patterns (PAMPs) like LPS and CpGA.
(TIF)
