# Supplementary figures and images for: Tolerance and Cross-Tolerance following Toll-Like Receptor (TLR)-4 and -9 Activation Are Mediated by IRAK-M and Modulated by IL-7 in Murine Splenocytes
Source: PLoS One. 2015 Jul 28;10(7):e0132921. doi: 10.1371/journal.pone.0132921 (PMC4517781; doi:10.1371/journal.pone.0132921)

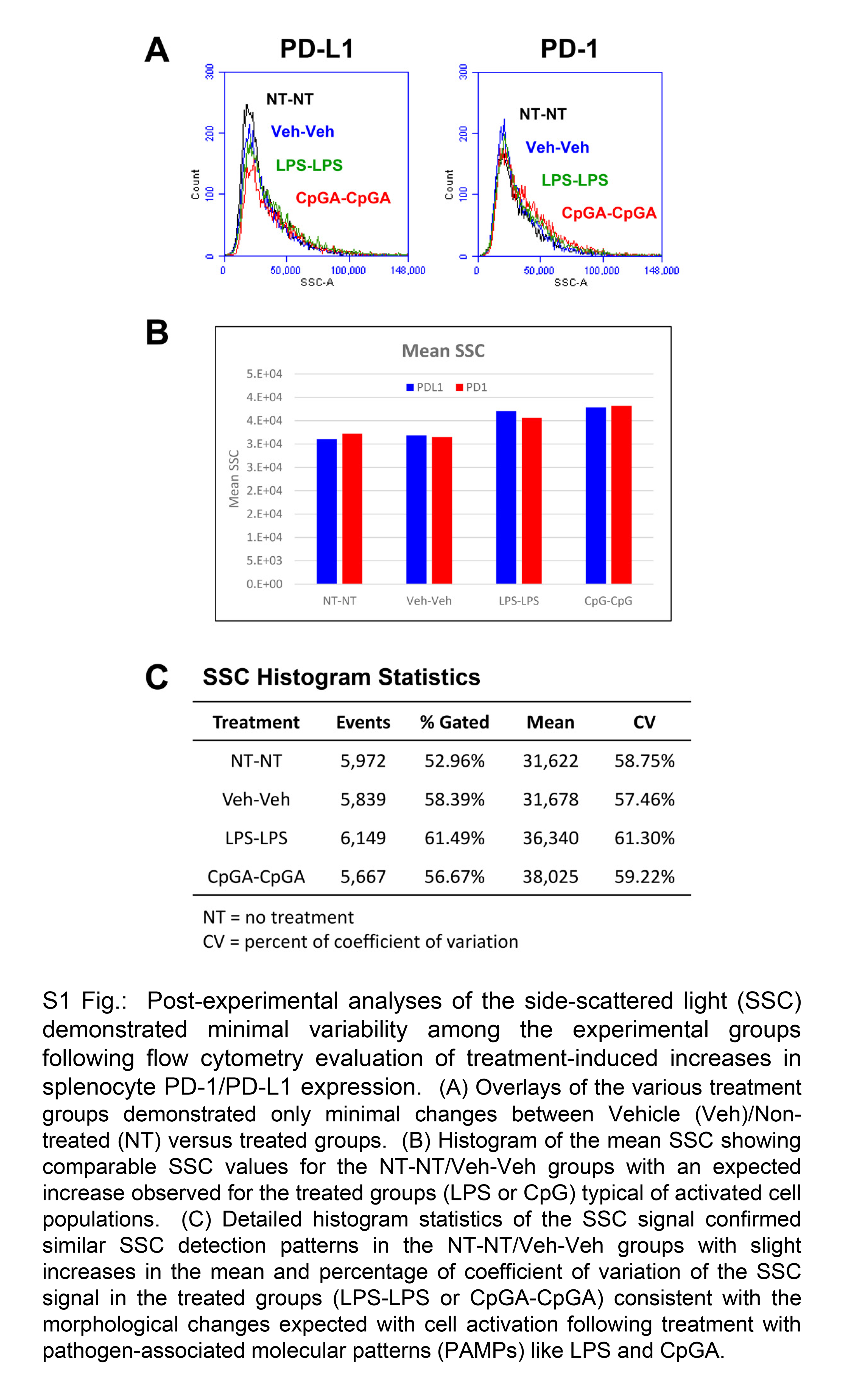

Supplement: S1 Fig — (A) Overlays of the various treatment groups demonstrated only minimal changes between Vehicle (Veh)/Non-treated (NT) versus treated groups. (B) Histogram of the mean SSC showing comparable SSC values for the NT-NT/Veh-Veh groups with an expected increase observed for the treated groups (LPS or CpG) typical of activated cell populations. (C) Detailed histogram statistics of the SSC signal confirmed similar SSC detection patterns in the NT-NT/Veh-Veh groups with slight increases in the mean and percentage of coefficient of variation of the SSC signal in the treated groups (LPS-LPS or CpGA-CpGA) consistent with the morphological changes expected with cell activation following treatment with pathogen-associated molecular patterns (PAMPs) like LPS and CpGA. (TIF) [file pone.0132921.s002.tif]
